# Rational Design of Molecularly Imprinted Polymers Using Quaternary Ammonium Cations for Glyphosate Detection

**DOI:** 10.3390/s21010296

**Published:** 2021-01-04

**Authors:** Mashaalah Zarejousheghani, Alaa Jaafar, Hendrik Wollmerstaedt, Parvaneh Rahimi, Helko Borsdorf, Stefan Zimmermann, Yvonne Joseph

**Affiliations:** 1 Institute of Electronic and Sensor Materials, Faculty of Materials Science and Materials Technology, TU Bergakademie Freiberg, 09599 Freiberg, Germany; Alaa.Jaafar@doktorand.tu-freiberg.de (A.J.); Parvaneh.Rahimi@esm.tu-freiberg.de (P.R.); Yvonne.Joseph@esm.tu-freiberg.de (Y.J.); 2 UFZ-Helmholtz Centre for Environmental Research, Department Monitoring and Exploration Technologies, 04318 Leipzig, Germany; helko.borsdorf@ufz.de; 3 Institute of Energy Process Engineering and Chemical Engineering, Chair of Reaction Engineering, Faculty of Mechanical, Process and Energy Engineering, TU Bergakademie Freiberg, 09599 Freiberg, Germany; Hendrik.Wollmerstaedt@iec.tu-freiberg.de; 4 Institute of Electrical Engineering and Measurement Technology, Department of Sensors and Measurement Technology, Leibniz University Hannover, 30167 Hannover, Germany; zimmermann@geml.uni-hannover.de

**Keywords:** imprinted polymer, quaternary ammonium cation, glyphosate, quartz crystal microbalance, design of experiment

## Abstract

Molecularly imprinted polymers have emerged as cost-effective and rugged artificial selective sorbents for combination with different sensors. In this study, quaternary ammonium cations, as functional monomers, were systematically evaluated to design imprinted polymers for glyphosate as an important model compound for electrically charged and highly water-soluble chemical compounds. To this aim, a small pool of monomers were used including (3-acrylamidopropyl)trimethylammonium chloride, [2-(acryloyloxy)ethyl]trimethylammonium chloride, and diallyldimethylammonium chloride. The simultaneous interactions between three positively charged monomers and glyphosate were preliminary evaluated using statistical design of the experiment method. Afterwards, different polymers were synthesized at the gold surface of the quartz crystal microbalance sensor using optimized and not optimized glyphosate-monomers ratios. All synthesized polymers were characterized using atomic force microscopy, contact angle, Fourier-transform infrared, and X-ray photoelectron spectroscopy. Evaluated functional monomers showed promise as highly efficient functional monomers, when they are used together and at the optimized ratio, as predicted by the statistical method. Obtained results from the modified sensors were used to develop a simple model describing the binding characteristics at the surface of the different synthesized polymers. This model helps to develop new synthesis strategies for rational design of the highly selective imprinted polymers and to use as a sensing platform for water soluble and polar targets.

## 1. Introduction

Glyphosate (N-(phosphonomethyl)glycine), as the most heavily used herbicide in the world, is nowadays recognized as a suspected human carcinogen and is blamed for the death of insects. However, glyphosate detection is very difficult due to its high polarity in aqueous solution and the lack of absorbance or fluorescence. Therefore, more enhanced glyphosate analysis is needed [1]. In comparison to highly sophisticated and expensive analytical instruments like liquid chromatography–mass spectrometry, sensors are relatively low-cost devices intended for use by inexperienced users for the simple monitoring of target molecules, biomolecules, and ions. Although a non-modified sensor has been used for glyphosate detection [2], sensors suffer generally from the low selectivity and sensitivity. To overcome these limitations, sensors were modified with different sorbent materials for sensitive and selective detection of glyphosate in water samples [3,4,5,6]. Nanocomposites are interesting materials, which have been used for modification of different sensors. A “copper-aluminum metal hydroxide doped graphene” nanocomposite was used to modify the surface of a glassy carbon electrode for glyphosate detection in water samples [7]. In another study, the surface plasmon resonance sensor was modified with different nanocomposites including chitosan/zinc oxide. This modified sensor enabled sensitive detection of glyphosate. Different parameters were optimized to increase the sensor selectivity. This helped to develop a sensor with a very good selectivity, but the competition of other molecules was still reported in aqueous systems [8]. In comparison to the non-selective sorbent materials, immunosorbents are highly specific towards a target molecule. Innovative methods have been developed in which antibodies and enzymes were used for selective detection of glyphosate [9]. Using a relatively complicated procedure, paramagnetic beads, modified with anti-glyphosate antibodies, were used for glyphosate detection using a competitive strategy [10]. In this study, modified beads were incubated with samples containing glyphosate. Afterwards, conjugate glyphosate-horseradish peroxidase (HRP) were added to fill the remained specific sites. The attached HRP was then used to oxidize a substrate in the presence of H_2_O_2_ and generate an electroactive product. Finally, the concentration of this product was measured using the electrochemical method and used for sensitive detection of glyphosate. Despite the high selectivity of immunosorbent materials, they are expensive, sensitive to the environmental conditions, and challenging to work with. Molecularly imprinted polymers (MIPs) are artificial selective receptors which imitate the behavior of antibodies [11]. Compared to immunosorbents, MIPs are known to have advantageous characteristics including low-cost, easy engineering, simplicity of production, potential reusability, physical/chemical stability, and their applicability for a wide range of targets [12,13,14]. MIPs are highly cross-linked co-polymers in which the recognition sites are imprinted for the special targets. Nowadays, even antibodies and enzyme conjugates in the enzyme-linked immunosorbent assay (ELISA) method could be replaced with imprinted polymers using new synthesis strategies like solid-phase synthesis of molecularly imprinted nanoparticles [15,16,17,18,19]. For glyphosate as a target molecule, different imprinted polymers were developed for direct detection (sensors) [20,21,22,23,24,25,26,27] or sample-preparation [28,29,30] purposes. The fundamental principles of MIP-technology can be found in other published review manuscripts [13,14,31]. In summary, before polymerization, the target molecule or ion (known as template) forms a complex with selected monomers through covalent, semi-covalent, or noncovalent interactions in the presence of a solvent (porogen). The complexed monomers are stabilized around the template using a cross-linker agent in a polymerization process. The imprinted cavities then remain within the polymer matrix even after removal of the template. These cavities therefore have a complementary size, shape, and spatial position of the functional groups towards the template [32,33,34]. 

Due to its inherent advantages (simpler synthesis strategy and the wide variety of commercially available functional monomers), the non-covalent approach is the most common used engineering method to design imprinted polymers [35]. Here, the intermolecular interactions, which are responsible to arrange the functional monomers around the template molecules, include hydrogen bonds, ionic interaction, van der Waals (VDW) forces, π-π interaction, and hydrophobic effects. While the hydrogen bond is the most used non-covalent interaction, oppositely charged ions can provide stronger intermolecular interaction (covalent bonds: ~500 kJ/mol; ionic interaction: up to 60 kJ/mole; hydrogen bond: up to 40 kJ/mole, charge-dipole interactions: up to 8 kJ/mole; dipole–dipole interaction: ~1 kJ/mol; VDW interactions: 0.1–1 kJ/mol) [35]. 

The most important challenge for traditional synthesis of glyphosate-imprinted polymer is its extremely low solubility in general porogens like chloroform and restricted number of suitable functional monomers. In most of the published manuscripts, glyphosate and selected functional monomers were dissolved in the large volumes of mixed porogens aimed to prepare the template-functional monomer complex based on hydrogen bonds [26,28]. Prasad et al. developed an interesting electrochemical sensor using derivatized glyphosate as the template [23]. A mixture of functional monomers, n-allylthiourea and 2-dimethyl aminoethyl methacrylate, was used to synthesize imprinted polymer for underivatized glyphosate [29]. To evaluate the electrostatic interactions and hydrogen bonds, 1-allyl-2-thiourea and methacrylic acid, were also used as functional monomers and different dummy templates instead of glyphosate [30]. Imprinting the polypyrrole for glyphosate by electropolymerization of pyrrole in the presence of glyphosate is another synthesis strategy which is reported by different authors [22,24,25]. Recently, Zouaoui et al. developed an interesting synthesis strategy in which chitosan-glyphosate was electrodeposited at the surface of a gold microelectrode [27].

We have already showed the applicability of ionic interaction for synthesizing a highly selective imprinted polymer for negatively charged acesulfame, a known anthropogenic marker, using (vinylbenzyl) trimethylammonium chloride (VBTA) as an efficient ion-pair reagent [36,37,38]. We believe that quaternary ammonium cations containing the vinyl group are among the most efficient and practical functional monomers, which could enable synthesizing selective imprinted polymers for negatively charged target molecules e.g., glyphosate.

In this study, a small pool of monomers was chosen containing three quaternary ammonium cations (QACs) in water. In MIP technology, the Job plot has been frequently used, as a standard method, to estimate the template-functional monomer stoichiometry [39]. In Job’s method, the total molar concentration of the template and a monomer is kept constant, but their mol fractions are changed and the relative signals are recorded to find the most efficient ratio. In our study, statistical design of the experiment (DOE) method was used to evaluate, systematically, the simultaneous interactions between three positively charged QACs and the negatively charged glyphosate. Afterwards, polymers were synthesized on the gold electrodes of quartz crystal microbalance sensors [40] using different template-functional monomers ratios (including optimized ratio). Synthesized polymers were finally evaluated using different characterization methods.

## 2. Materials and Methods

The chemicals used in this study were ethylene glycol dimethacrylate (EGDMA) (CAS No. 97-90-5), 2,2′-azobis(2-methylpropionitrile) (AIBN) (CAS No. 78-67-1), (3-acrylamidopropyl)trimethylammonium chloride solution (75 wt.% in H_2_O) (CAS No. 45021-77-0), [2-(acryloyloxy)ethyl]trimethylammonium chloride solution (80 wt.% in H2O) (CAS No. 44992-01-0), diallyldimethylammonium chloride (CAS No. 7398-69-8), glyphosate (CAS No. 1071-83-6), (aminomethyl)phosphonic acid (AMPA) (CAS No. 1066-51-9), dichlorodimethylsilane (DCDMS) (CAS No. 75-78-5), 9-fluorenylmethoxycarbonyl chloride (Fmoc-Cl 97%) (CAS No. 28920-43-6), dimethyl sulfoxide (DMSO) (CAS No. 67-68-5), and borax anhydrous (CAS No. 1330-43-4). They were obtained from Sigma-Aldrich. Chloroform (CAS No. 67-66-3), acetonitrile (CAS No. 75-05-8), ethanol (CAS No. 64-17-5), sulfuric acid 95% (CAS No. 7664-93-9), hydrogen peroxide solution 30% (CAS No. 7722-84-1) were bought from MERCK (Darmstadt, Germany). Allyl mercaptan (>70.0%) (CAS No. 870-23-5) was obtained from Tokyo Chemical Industry Co., Ltd. (TCI; Tokyo, Japan). 

The stock standard solutions of glyphosate and AMPA were prepared in distilled water at a concentration of 1000 mg L^−1^ and stored in the refrigerator. Other standard solutions were daily prepared via the dilution of the stock solution using pH adjusted deionized water. All standards were prepared in polypropylene (PP) bottles to prevent any loss of glyphosate and AMPA on glass surfaces.

Borate buffer solution (BBS; pH~9.4) was prepared in distilled water. The standard solution of Fmoc-Cl was freshly prepared daily in acetonitrile at a concentration of 2000 mg L^−1^ and used for derivatization of glyphosate and AMPA in 2 mL PP centrifuge tubes. However, derivatized samples must be then transferred into 1.5 mL autosampler glass vial for further high-performance liquid chromatography (HPLC) analysis. Therefore, in order to prevent the surface adsorption of glyphosate and AMPA molecules to the vials, glass vials were previously silanized using 5% DCDMS in hexane. Glass vials were remained in contact with 5% DCDMS in hexane for 10 min. After that, vials were washed with hexane and acetone before being dried [41]. Caution! We warn the readers of the hazards of working with highly flammable DCDMS. It reacts vigorously with water to generate hydrogen chloride. 

The central composite design (CCD) as a widely accepted experimental plan was used to study the entire parameters of the processes with limited number of experiments. In this investigation, the freedom degrees satisfied by 16 runs and each run has a different combination of the quaternary ammonium cations amounts at constant concentration of glyphosate. The factors and their levels are demonstrated in Table 1.

In order to accomplish the DOE, 1 mL of glyphosate standard solution in distilled water was mixed with 60 µL of BBS (pH~9.4) to ionize the glyphosate molecules. Then, quaternary ammonium cations (A, B, and C) were added according to the defined 16 runs in Table 2. The prepared mixture was stirred gently for 30 min. After that, 5 mL chloroform was added to the mixture and stirred vigorously for 60 min. After completing the 60 min stirring, 50 µL of water phase was separated and mixed with 2000 µL of distilled water and stored in refrigerator for further HPLC-UV analysis. Additionally, 50 µL of chloroform phase was also separated and mixed with 10 mL of distilled water and stored in refrigerator for further HPLC-UV analysis.

The idea of adding chloroform comes from our previously manuscript [38] in which negatively charged acesulfame in chloroform was detected (with HPLC-MS-MS) by adding a phase transfer agent into water sample. Here, we want to see if highly water-soluble glyphosate could be also detected in organic phase. 

For HPLC-UV analysis, samples were first derivatized with Fmoc-Cl. To this aim, 1 mL of each stored sample was mixed with 60 µL BBS and then 200 µL Fmoc-Cl solution (2000 mg L^−1^ in acetonitrile and prepared freshly each day) was added. The mixture was shaken and rest for at least 1 h before analyzing with HPLC-UV.

The standard analysis was performed using an HPLC instrument HPLC Dionex Ultimate 3000 (Dionex, Part of Thermo Fisher Scientific, Dreieich, Germany) equipped with a binary pump, a membrane degasser, an autosampler and an Ultimate 3000 photodiode array detector (DAD). The chromatographic separation was performed by a Kinetex 5 µ C18 100A column (Phenomenex LTD, Aschaffenburg, Germany) with 250 mm length and 4.6 mm I.D (5 µm particle size). The eluent consisted of distilled water (pH 7) and acetonitrile at 0.5 mL min^−1^. The column temperature was constant at 30 °C. Then, 20 µL of samples were injected automatically. Gradient elution was chosen to separate all compounds. At the beginning of the analysis, the mobile phase consisted of 80:20 distilled water:acetonitrile mixture. Then, the organic percentage of the mobile phase was increased gradually to 90% over 5 min and then, it was decreased again to 20% over 5 min and maintained constant at 20% until the end of analysis (15 min). For our evaluation purposes, four wavelengths were used as following: 206, 210, 220, and 250 nm (bandwidths: 4 nm). Regularly blank and glyphosate/AMPA standard analyses were carried out to check carryover effects and instrument performance.

A Topometrix TMX-2010 atomic force microscope (AFM) was used for topographic imaging of the gold surfaces. Standard silicon cantilevers were used which were coated with Si_3_N_4_ (MikroMasch CSC12/Si_3_N_4_). A force constant of 0.03 N m^−1^ was applied with resonant frequency of 10 kHz and radius of curvature <20 nm. Topography and lateral force images were simultaneously measured at a scan rate of 1–2 Hz under ambient laboratory conditions.

TENSOR II from Bruker (Leipzig, Germany) was used for Fourier-transform infrared spectroscopy (FTIR) measurements. A built-in standard measurement procedure with A513/Q variable angle reflection accessory (30°, 50° reflection angle) was used under ambient laboratory conditions (atmospheric correction of spectra) to collect infrared spectra.

A drop shape analyzer from KRÜSS GmbH (Hamburg, Germany) was used to evaluate the contact angle of 2 µL water drop on the sensor surfaces using sessile drop technique.

Low-pressure plasma systems from Diener electronic GmbH (Ebhausen, Germany) was used to produce oxygen plasma for plasma treatment of surfaces.

X-ray photoelectron spectroscopy (XPS) was performed on a Thermo Fisher Scientific ESCALAB 250Xi spectrometer using a monochromatic Al Kα X-ray source (1486.6 eV) operated at 250 W (14.6 kV, 17 mA). A pass energy of 20 eV as well as an energy step size of 0.1 eV were used for the collection of high resolution spectra of Au4f, C1s, Cl2p, N1s, O1s, P2p and S2p. The pressure of the system was about 5 × 10^−10^ mbar, which rose to approximately 2 × 10^−7^ mbar with the use of combined electron/Ar-ion beam charge compensation. Data processing was carried out using the CasaXPS software package (http://www.casaxps.com). Before analysis, the binding energy calibration was performed using the Au-4f_7/2_ line to 84.0 eV. The fits of the high resolution spectra were performed by using Voigt-profiles (70% Gauss/30% Lorentz) and fixed binding energies of the expected species.

Gold coated 5 MHz QCM sensor crystals were purchased from MicroVacuum (Budapest, Hungary). A commercial microfluidic quartz coating cell from QSense with laminar flow and a volume of 140 μL (40 μL above the quartz) was used for flow injection analysis (FIA). An automated FIA method, developed in our institute [42], was used to measure water samples. In summary, a peristaltic pump (Ismatec, ISM935C) was used to adjust the liquid flows, which flow through the cell. A computer-controlled six-way valve (Hamilton MVP) was also used to switch between the samples and blank at the desired timetable.

**Figure 1:**
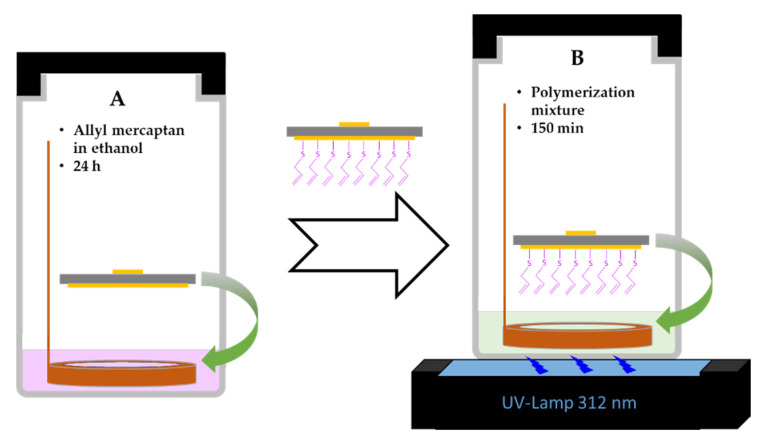
Schematic representation of the used procedure for the surface modification of quartz crystal microbalance (QCM) sensors. (**A**) Preparation of a self-assembled monolayer of allyl mercaptan on the gold surface and (**B**) following polymerization inside the polymerization precursors.

A VL-6LMUV-lamp (6 W, 312 nm) was used for the synthesis of the imprinted polymers. The polymers were synthesized at the gold surface of QCM sensors. To this aim, the gold surface (Au) of QCM sensors was pre-modified with allyl mercaptan (Au-AM) and used for further surface polymerization. Surface modification of QCM sensors with allyl mercaptan was adopted from Diltemiz et al. [40]. In summary, gold surfaces were cleaned with piranha solution (1:3, 30% H_2_O_2_:95% H_2_SO_4_) for 30 s and oxygen plasma for 10 min. Caution! Extreme precaution must be taken during preparation and application of piranha solution. It is extremely corrosive and reacts vigorously with organic compounds. After cleaning, QCM sensors were immersed into 20 mL absolute ethanol that contained 50 µL allyl mercaptan (>70.0%) and maintained for 24 h (Figure 1). After this preliminary modification, QCM sensors were washed with ethanol and acetone and stored in ethanol for further modifications.

Allyl mercaptan-modified QCM sensors were then further modified with different types of polymers. To this aim, ingredients for the polymerization mixture were weighed in a PP centrifuge tubes. Then, 1 mL distilled water, 60 µL BBS and 1 mL DMSO were added and stirred gently for 30 min. After that, each polymerization mixture was added into a glass vial that contained 10 mg AIBN as initiator, 774 µL DMSO and 226 µL EGDMA as cross-linker. As shown in Figure 1, allyl mercaptan-modified QCM sensors were then immersed into the polymerization mixtures using a Teflon ring by which the modified gold surface of the QCM sensors was about 3–5 mm away from the UV-lamp. Finally, each glass vial was degassed with argon for 10 min and polymerized using a UV lamp at 312 nm for 150 min. After synthesis of the polymers, they were completely washed with acetone, ethanol, and distilled water. 

## 3. Results and Discussion

### 3.1. Functional Monomers and the Glyphosate-Functional Monomers Ratio Optimization Using Design of Experiment (DOE)

**Figure 2:**
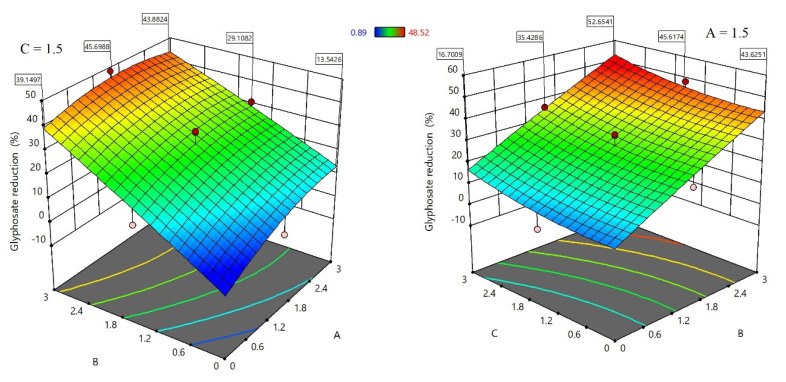
Effect plot of quaternary ammonium cations on glyphosate reduction.

Glyphosate is a polar organophosphorus compound, which is highly soluble in water, especially at higher pHs. In the neutral and alkaline conditions, glyphosate can be easily deprotonated and produce different negatively charged ions (Figure S1) [43]. Therefore, it is a suitable model molecule for our investigations. In the first step, three cations were selected, as shown in Table 1, to evaluate the functionality of quaternary ammonium cations (QACs). Both glyphosate and cations were dissolved in alkaline water and their interactions were systematically evaluated using the statistical DOE method. Table 2 shows the amount of glyphosate (µg) which was founded in water phase. Without adding quaternary ammonium cations (A, B, and C), 19 µg glyphosate was expected to be detected. Using this value, the percent of glyphosate-reduction for each row of Table 2 was calculated and used for further DOE optimization. The analysis of variances (ANOVA) and the effect plots are shown in Table 3 and Figure 2, respectively. 

Interestingly, the amounts of glyphosate in chloroform phases were negligible. The reasons could be either the lower concentrations of used glyphosate and complexation agents in water phase or the less-sensitive HPLC-UV analysis method, which was used in this study.

ANOVA results indicated that the addition of quaternary ammonium cations have a significant role in reducing the amount of detectable glyphosate in the water solution. However, [2-(Acryloyloxy)ethyl] trimethylammonium chloride (B) is the dominant factor with highest contribution in this process compared to other studied cations, while the effect of the square and the interactions between the cations are negligible. As shown in Figure 2, introduction of B cation causes a sharp and linear increase in the reduction of glyphosate content. The same behavior but with lower intensity is noted with the introduction of C cation. Interestingly, the addition of A cation at high concentrations up to 3 mmol has a negative effect and led to a decrease of the evaluated signal (glyphosate-reduction). This suggests using lower concentration of A to obtain the maximum glyphosate-reduction.

The relationship between the quaternary ammonium cations and the percentage of glyphosate-reduction was modeled by the quadratic regression model. The model and its coefficient of determination is presented in the equation below:

Glyphosate reduction (%) = −5.27291 + 11.25971 A + 14.94071 B − 0.590954 C − 1.36222 A ∗ B + 0.063333 A ∗ C + 0.017778 B ∗ C − 1.90268 A^2^ − 0.264904 B^2^ + 1.19732 C^2^.

R^2^ = 93.68%.

The above model is capable to predict the percentage of glyphosate-reduction for any collection of quaternary ammonium cations concentrations within the conducted experiment range. The mismatch between the experimental and predicted results based on this model is shown in Figure S2. This figure indicates that the developed mathematical model could efficiently predict the reduction of glyphosate, under the given experimental conditions. The desirability function approach has been utilized to search for the best set of optimum combinations of quaternary ammonium cations concentrations that result in the maximization of the glyphosate reduction. For 1 mmol glyphosate, the obtained ratio for cations, as A = 2 mmol, B = 3 mmol, and C = 3 mmol, could provide the highest percent glyphosate-reduction (53.43%) with a desirability of 0.815 as demonstrated in Figure S3.

### 3.2. Polymerization and Characterization

After interactions evaluation of the glyphosate and selected cations, two different imprinted polymers and their related non-imprinted polymers were synthesized using (i) optimized cations ratio as glyphosate:A:B:C (1:2:3:3 mmol) and (ii) glyphosate:B (1:3 mmol). Cation B was selected to synthesize the second polymer, because it was found as the most efficient cation, which could interact with glyphosate. Table 4 shows the used polymerization mixtures for synthesizing imprinted polymers (MIP-B, MIP-ABC) and non-imprinted polymers (NIP-B, NIP-ABC). In this study, we used the very common template-crosslinker ratio (1:20) to start our evaluation. However, this ratio can also be evaluated in further investigations. The polymers were synthesized at the gold surface of QCM sensors, as described in the section Materials and Methods. 

Polymer-modified QCM sensors (MIP-B, NIP-B, MIP-ABC, and NIP-ABC), together with a non-modified QCM (Au) and a QCM sensor which was just modified with allyl mercaptan (Au-AM), were further characterized with AFM and contact angle methods. 

**Figure 3:**
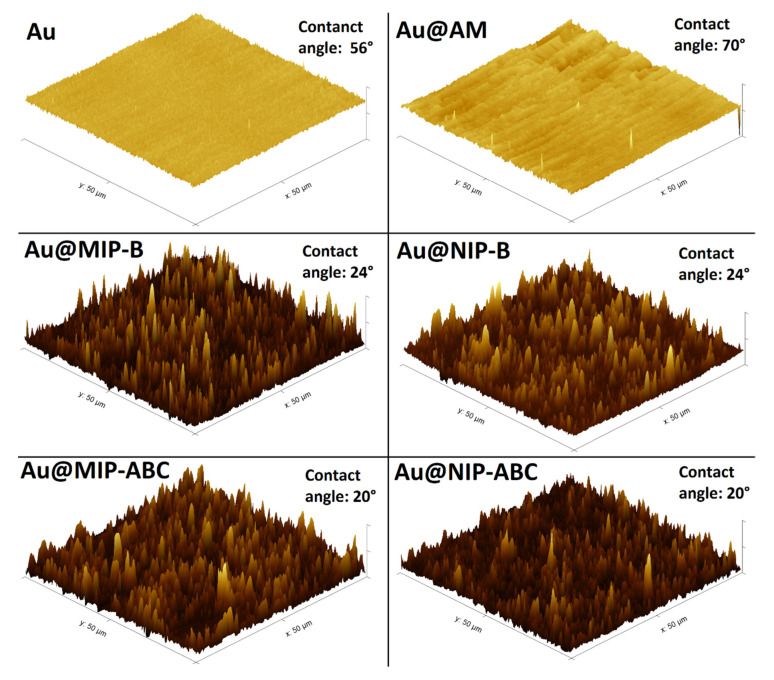
AFM 3D images and contact angle measurements of the non-modified (Au) and modified QCM sensors.

Figure 3 shows the obtained AFM 3D images of the non-modified and modified sensors. Contact angle measurements were repeated ten times for each sensor with water drop and the obtained averages are represented in Figure 3. AFM 2D images and the height profiles for the corresponding lines drawn in AFM images are shown in Figure S4. 

The changes in the resonance frequency of QCM sensors, after modifications with polymers, were used to calculate the layer’s height of the coatings as the following: MIP-B: 0.4 µm, NIP-B: 0.9 µm, MIP-ABC: 0.5 µm, and NIP-ABC: 0.8 µm. The mean contact angle results show that the wettability of the gold surface was reduced after its modification with allyl mercaptan (56°→70°). After polymerization, the wettability of all modified surfaces was strongly increased, probably due to the addition of positively charges to the surfaces. Obtained results for MIP-B and NIP-B are in the same range. However, MIP-ABC and NIP-ABC have slightly lower contact angles presumably due to more cations, which are available in the structure of the polymer matrix. These results show that the bare gold surface of the QCM sensors were successfully modified with allyl mercaptan and polymers.

**Figure 4:**
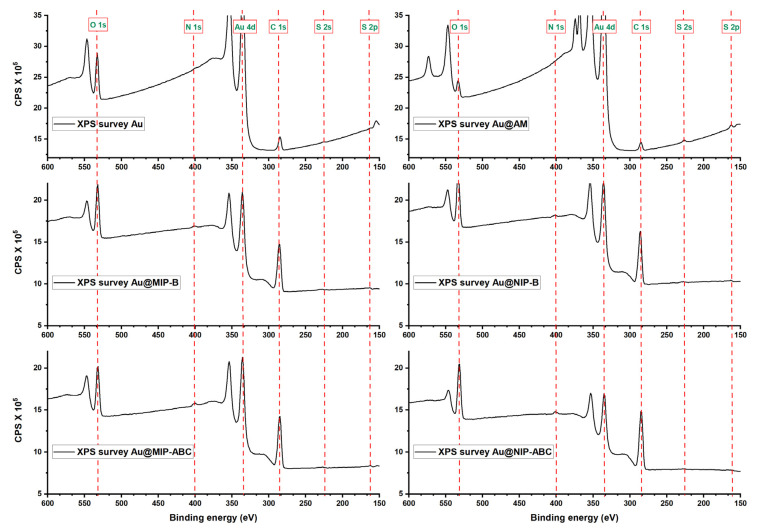
XPS survey spectra of all evaluated sensors (from 150 to 600 eV).

FTIR spectra of the evaluated sensors are shown in Figure S5. FTIR spectra for synthesized MIPs and NIPs show strong peaks at the 2840–3000 cm^−1^ region which could be attributed to C-H stretching. The Au@AM sensor provides weak peaks in the same region, which could be attributed to the attached allyl mercaptan. A very weak peak is also available at 2969 cm^−1^ for the not modified sensor (Au) which could be related to the small amounts of contaminations available at the gold surfaces, even after complete washing with piranha solution and oxygen plasma. MIPs and NIPs modified sensors provide also medium peaks at 1377 and 1471 cm^−1^ which could be attributed to C-H bending [44]. Unfortunately, the FTIR method could just detect C-H bonds at the gold surfaces. Therefore, elemental compositions of the sensors were further evaluated using the sensitive XPS method. Figure 4 shows the XPS survey of all evaluated sensors (this figure zoomed in to 150–600 eV range to highlight the smaller peaks for S 1s, S 2P, and N 1s. The complete range can be seen in Figure S6). 

**Figure 5:**
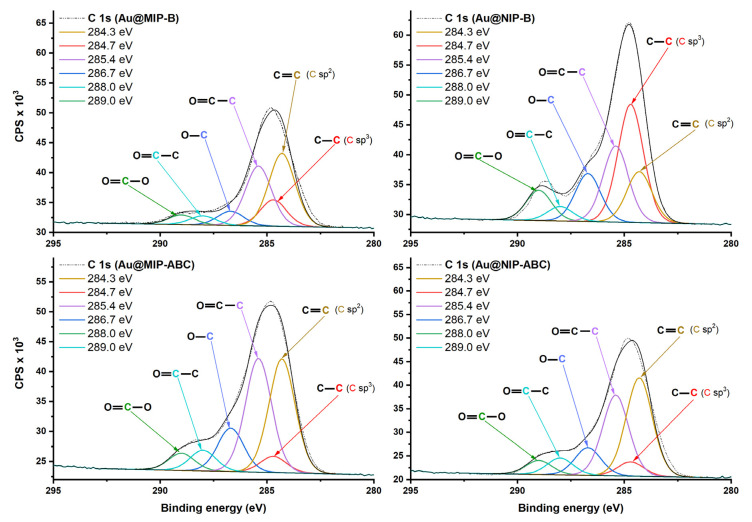
C 1s high-resolution XPS spectra of sensors modified with imprinted and non-imprinted polymers.

**Figure 6:**
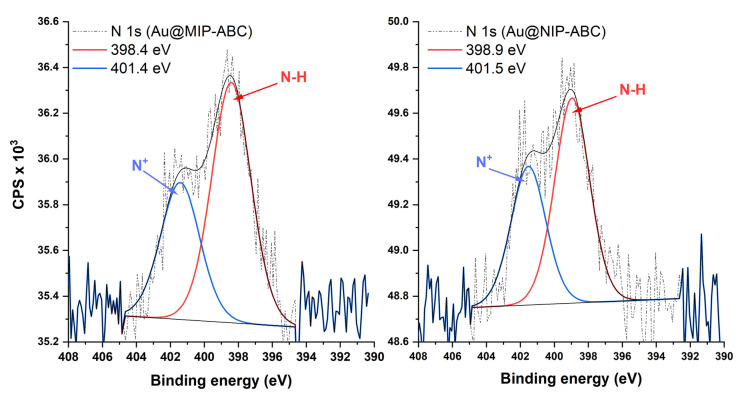
N 1s high-resolution XPS spectra of sensors modified with imprinted and non-imprinted polymers.

Figure 4 shows that two small peaks were created at 162.49 eV (S 2p) and 226.35 eV (S 2s) after surface modification with allyl mercaptane. These two peaks could be hardly detected for MIP and NIP modified QCM, probably due to the covering of allyl mercaptane layer with polymers showing the limitation in the information depth of the method. Strong C 1s peaks were detected for the modified sensors with polymers. The C 1s high-resolution spectra (Figure 5) show six peaks which are attributed to different carbon atoms within the structure of polymers matrices [45]. The O 1s high-resolution spectra (Figure S7) show two peaks at ~532 and ~533.5 eV which are attributed to oxygen atoms in O=C and O-C, respectively. Besides the strong C 1s and O 1s peaks, the weak N 1s peaks were detected in XPS survey spectra of polymer-modified sensors (Figure 4). N 1s peaks for MIP-ABC and NIP-ABC were relatively stronger (probably due to higher concentration of nitrogen within the polymer matrix) and could be further evaluated using high-resolution spectra (Figure 6). These spectra show clearly two peaks at 398.69 ± 0.3 eV and 401.48 ± 0.1 eV which can be attributed to NR_3_ and NR_4_^+^, respectively [46].

### 3.3. Investigation of Sensor Application of Designed Polymers Using QCM Measurements

**Figure 7:**
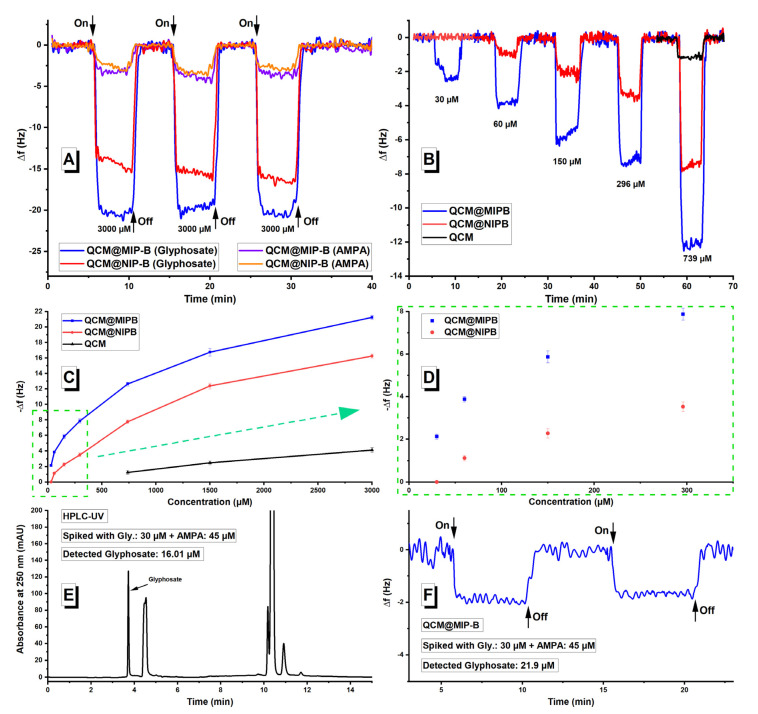
QCM@MIP-B, QCM@NIP-B, QCM signals (**A**) for glyphosate and (aminomethyl)phosphonic acid (AMPA) both at 3000 µM, (**B**) for glyphosate at 30, 60, 150, 296, and 739 µM, (**C**) for glyphosate at concentration rage 30–3000 µM, and (**D**) 30–296 µM, (**E**) real sample analysis using standard HPLC-UV detector and (**F**) real sample analysis using QCM@MIP-B sensor.

**Figure 8:**
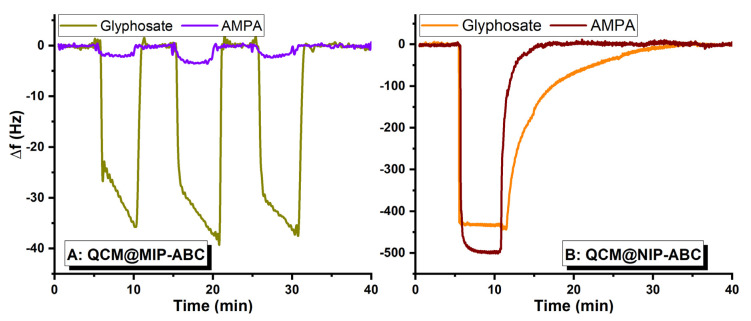
(**A**) QCM@MIP-ABC and (**B**) QCM@NIP-ABC signals for glyphosate and AMPA both at 3000 µM.

After the structural characterization of the polymers, the binding characterization of glyphosate was evaluated at the surface of imprinted and non-imprinted polymers. Glyphosate and its primary degradation product, AMPA, can be hardly detected with a non-modified QCM sensor at the concentration ranges below 700 µM. Due to the low sensitivity of non-modified QCM sensors towards glyphosate and AMPA, we had to start our preliminary evaluation at high concentration (3000 µM) to evaluate the functionality of the sensors. Figure 7A and Figure 8 show the obtained results at 3000 µM for all the modified QCM sensors. In Figure 7A, comparison of the QCM@MIP-B and QCM@NIP-B signals for glyphosate shows a notable difference, while the AMPA signals were nearly the same. MIP and NIP difference for glyphosate shows that the glyphosate cavities were imprinted within the polymer during polymerization process. However, during the polymerization, imprinted sites with different affinity for the template and the non-selective sites are usually formed in the molecularly imprinted polymers. It is known that MIPs show lower selectivity at high analyte concentrations. In contrast, selectivity increases rapidly to very high values at low analyte concentrations. This behavior arises from this fact that the low affinity and low selectivity sites are generally sampled at higher concentrations and the high affinity, high selectivity sites are mostly sampled at lower concentrations [47]. 

Therefore, selectivity of synthesized MIP-B was further evaluated at lower concentrations. To this aim, QCM@MIP-B and QCM@NIP-B were used to detect glyphosate at lower concentrations (Figure 7B). The results clearly show that MIP and NIP differences are increased at lower concentrations, which prove the presence of recognition sites for glyphosate within the MIP-B polymer matrix.

Figure 7A shows also that both MIP-B and NIP-B adsorb nearly the same amount of AMPA molecules. AMPA has many similarities to glyphosate as the parent molecule, whether the structure or functional groups, but it is a smaller molecule (M_AMPA_ = 111.04 g mol^−1^ and M_Glyphosate_ = 169.07 g mol^−1^). Therefore, AMPA could not be fitted into the recognition sites and can be adsorbed just by non-selective sites as it happens in the non-imprinted polymer. QCM measurements for a concentration range from 30 to 3000 µM are shown in Figure 7C. MIP-B and NIP-B difference at lower concentrations is highlighted in Figure 7D. As a proof of concept, the QCM@MIP-B sensor was also used to detect glyphosate in a real water sample in the presence of AMPA. To this aim, the water sample from a river (Freiberg, Germany) was first filtered to remove the suspended particles and then spiked with glyphosate and AMPA. Figure 7E,F show the obtained results for real sample analysis using the standard HPLC-UV method in comparison to the developed QCM@MIP-B sensor. Despite the successful results, sensitivity of the modified QCM@MIP-B sensor is not high enough for direct trace detection of glyphosate. One important reason for the low sensitivity is the implementation of just [2-(acryloyloxy)ethyl]trimethylammonium chloride (B), as positively charged functional monomer, in the structure of MIP-B. 

Figure 8A shows that the QCM@MIP-ABC, having three positively charged monomers in the polymer’s structure, could adsorb about twice as much as QCM@MIP-B adsorbs during 5 min sample injection. Additionally, it shows that the QCM@MIP-ABC has nearly the same adsorption behavior towards AMPA as it was seen for the QCM@MIP-B sensor. Interesting results were obtained for the QCM@NIP-ABC sensor (Figure 8B). NIP-ABC was synthesized using the same polymerization precursors as MIP-ABC, but without the template molecules (template molecules were also washed away from MIP-ABC after polymerization). Comparison of Figure 8A,B shows that the template addition changes considerably the polymer’s structure, whereupon the imprinted polymer adsorbs selectively the target molecules and loses the sensitivity (This effect was not seen for MIP-B and NIP-B). While the QCM@NIP-ABC signal for glyphosate was increased up to 13.5 times more than the QCM@MIP-ABC, the AMPA signal was increased 200 times. 

Additionally, complete elution of glyphosate from QCM@NIP-ABC needs a prolonged time (≈35 min), but AMPA could be easily washed away (≈7 min). Recovery times for other evaluated modified sensors are about 1 min. These results show that the NIP-ABC polymer can adsorb glyphosate molecules via a kind of strong interaction which can be remained, even at longer elution time. 

**Figure 9:**
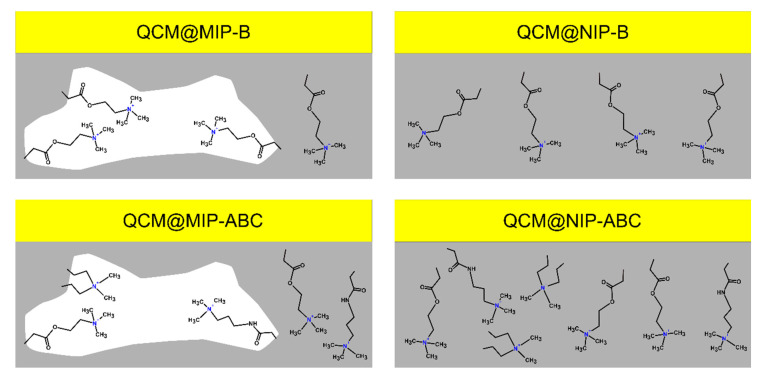
A simple model describing the main interactions, which control the glyphosate adsorption by different synthesized polymers.

Using these interesting data, we developed a simple model describing the interactions, which presumably control the glyphosate adsorption by different synthesized polymers (Figure 9). All polymers were synthesized in alkaline condition (pH~9), in which the glyphosate ion molecule (II) is the dominant ion. Enhanced glyphosate adsorption by QCM@MIP-ABC, in comparison to QCM@MIP-B, indicates that the synthesized polymer using three cations (A, B, and C) at the optimized ratio can adsorb the template molecules more efficiently, as predicted by the DOE method. These observations suggest that using different types of positively charged monomers at an optimized ratio can provide an adsorbent, which works much more efficient than a polymer, which is synthesized with just one type of monomer, even at a desired ratio. In this manuscript, glyphosate–monomers interactions were evaluated using a relatively simple statistical method. However, a more sophisticated statistical method, e.g., the neural network approach [48], can be used later for the precise evaluation of more complex interactions between a desired target and the large numbers of functional monomers.

Highly increased signals in QCM@NIP-ABC measurements show that the glyphosate and AMPA molecules could strongly adsorb to the surface of the NIP-ABC polymer. This suggests that the negatively-charged glyphosate molecule, as a known chelating agent [49], could strongly interact with randomly-distributed and stabilized cations at the surface of the NIP-ABC polymer. This effect could also explain the prolonged time, which is needed for complete washing of glyphosate molecules. On the other hand, AMPA molecules, with lower negative sites and molecular flexibility, can be adsorbed in a larger amount by the NIP-ABC polymer, but are more easily washed away. These results show that the polymer affinity can be manipulated by not only the creation of recognition sites but also changing the polymer composition. Shea’s group have shown that the protein corona of hydrogel nanoparticles (NPs) can be tuned by controlling the chemical composition of the NPs [50,51,52]. They have even studied different positively charged functional groups, as polymerization precursors, to synthesize cationic-functionalized polymer NPs with different affinities to fibrinogen [53]. Here, they synthesized different NPs, each incorporating either N-(3-methacrylamidopropyl) guanidinium chloride containing guanidinium group or N-(3-aminopropyl) methacrylamide hydrochloride containing primary amino group or (3-acrylamidopropyl)trimethylammonium chloride containing quaternary ammonium group. In our study, the synthesized NIP-ABC polymer layer increased the adsorption of glyphosate and AMPA with different affinities. In these types of polymers, affinities come from the nature of the polymer and not from the imprinted recognition sites. 

Now the question is; how can we synthesize recognition sites containing three monomers, while mitigating the creation of non-selective sites? Synthesizing the polymer in the pool after complexation could result in creation of non-selective sites. Therefore, separation of the prepared template-functional monomers complexes, before polymerization, from the pool of monomers is of great interest. This strategy is still a great challenge and we are working on it. This complexation strategy and following complex-separation and polymerization, are expected to provide polymers with high affinity towards the template molecules. For further investigations, in addition to polymer improvement, the 10 or 20 MHz quartz sheets could help to increase the sensitivity of modified sensors. Furthermore, dual electrodes on the same quartz sheet could be applied to compensate the temperature influences [54]. 

## 4. Conclusions

Imprinted polymers have been frequently used as innovative artificial selective sorbent materials for modification of different sensors. To synthesize selective imprinted polymers, functional monomers and their preliminary interactions with the target molecule (template), before polymerization, have significant roles. These are of vital importance for highly polar template molecules, as the numbers of suitable functional monomers for polar templates are restricted. In this manuscript, highly water soluble and negatively charged glyphosate was used, as an important model compound, to evaluate the functionality of positively charged quaternary ammonium cations (QACs) for synthesizing imprinted polymers. In MIP technology, the Job plot has been generally used to estimate the optimized stoichiometry of the template and one selected functional monomer. In our study, glyphosate itself selects the suitable functional monomers and the optimized ratio. To this aim, glyphosate was dissolved in a pre-designed small pool of three different QACs and their interactions were evaluated using the statistical DOE method (In the future, using the pools with larger numbers of functional monomers could be beneficial). In order to evaluate the sensing characterization of polymers, they were then synthesized at the gold surface of quartz crystal microbalances using different glyphosate-QACs ratios. The MIP-modified sensor could selectively and sensitively detect glyphosate in comparison to the NIP-modified and bare sensors. According to the obtained results, new synthesis procedures can be developed to improve the sensing features of MIP-modified sensors, which are still under development in our group. 

## Figures and Tables

**Table 1:** The controlled factors and their levels.

Factors		Notation	Units	Levels		
(3-Acrylamidopropyl)trimethylammonium chloride	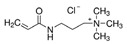	A	mmol	0	1.5	3
[2-(Acryloyloxy)ethyl]trimethylammonium chloride	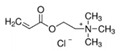	B	mmol	0	1.5	3
Diallyldimethylammonium chloride	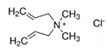	C	mmol	0	1.5	3
Glyphosate	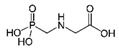	D	mmol	1	1	1

**Table 2:** Experimental results for design of experiments (16 runs).

No.	A	B	C	Found Glyphosate (µg)	SD	Glyphosate-Reduction (%)
1	3	0	3	14.52	0.84	23.57
2	3	1.5	1.5	13.12	2.14	30.94
3	0	1.5	1.5	16.77	2.58	11.73
4	0	0	3	18.35	0.48	3.42
5	0	3	3	9.78	1.87	48.52
6	3	3	0	10.81	1.39	43.10
7	1.5	1.5	3	12.18	1.46	35.89
8	3	0	0	17.02	0.40	10.42
9	0	3	0	12.20	1.32	35.78
10	1.5	1.5	0	15.06	1.15	20.73
11	0	0	0	18.83	0.60	0.89
12	1.5	1.5	1.5	12.78	1.54	32.73
13	1.5	1.5	1.5	12.68	1.72	33.26
14	1.5	0	1.5	18.66	1.07	1.780
15	3	3	3	10.19	2.35	46.36
16	1.5	3	1.5	9.83	1.80	48.26

**Table 3:** ANOVA table.

Source	^1^ DF	^2^ SS	^3^ MS	F-Value	*p*-Value	^4^ Cont.%
Model	9	3957.3	439.7	9.88	0.0057	93.68
A	1	292.14	292.14	6.56	0.0428	6.91
B	1	3310.22	3310.22	74.36	0.0001	78.36
C	1	219.4	219.4	4.93	0.0682	5.19
AB	1	75.15	75.15	1.69	0.2415	1.78
AC	1	0.1624	0.1624	0.0036	0.9538	0
BC	1	0.0128	0.0128	0.0003	0.987	0
A^2^	1	48.32	48.32	1.09	0.3376	1.14
B^2^	1	0.9366	0.9366	0.021	0.8894	0.02
C^2^	1	19.13	19.13	0.4298	0.5364	0.45
Residual	6	267.09	44.51			6.32
Lack of Fit	5	266.95	53.39	380.13	0.0389	6.32
Pure Error	1	0.1405	0.1405			0
Total	15	4224.39				100

^1^ DF: degrees of freedom, ^2^ SS: sum of squares, ^3^ MS: Mean square, ^4^ Cont.%: contribution percentage (ss parameter/ss total).

**Table 4:** Polymer precursors used to synthesize imprinted and non-imprinted polymers.

	Glyphosate (g)	A (g)	B (g)	C (g)	Porogen ^1^ (µL)	EGDMA ^2^ (µL)	AIBN ^3^ (mg)
MIP-B	0.0102	-	0.0477	-	2834	226	10
NIP-B	-	-	0.0440	-	2834	226	10
MIP-ABC	0.0106	0.0336	0.0453	0.0285	2834	226	10
NIP-ABC	-	0.0320	0.0456	0.0293	2834	226	10

^1^ Porogen: Distilled water + dimethylsulfoxid; ^2^ EGDMA: Ethylene glycol dimethylacrylate; ^3^ AIBN: 2, 2′-Azobis(2-methylpropionitrile).

## Data Availability

The data presented in this study are available in article and supplementary material.

## Supplementary Materials

The following are available online at https://www.mdpi.com/1424-8220/21/1/296/s1, Figure S1: Different glyphosate and AMPA ions at different pHs (https://chemicalize.com), Figure S2: Actual vs. Predicted plot for the developed model, Figure S3: Desirability ramp of optimization, Figure S4: AFM 2D images and the height profiles for the corresponding lines drawn in AFM images, Figure S5: FTIR spectrums of the evaluated sensors, Figure S6: XPS survey spectra of all evaluated sensors (from 0 to 1350 eV), Figure S7: O 1s high-resolution XPS spectra of sensors modified with imprinted and non-imprinted polymers.
