# Supplementary material for: Rational Design of Molecularly Imprinted Polymers Using Quaternary Ammonium Cations for Glyphosate Detection
Source: Sensors (Basel). 2021 Jan 4;21(1):296. doi: 10.3390/s21010296 (PMC7795498; doi:10.3390/s21010296)
Supplement: Supplementary file 1 [file sensors-21-00296-s001.pdf]

Supplementary Materials

Rational Design of Molecularly Imprinted Polymers Using Quaternary Ammonium Cations for Glyphosate Detection

Mashaalah Zarejousheghani [1,2,*], Alaa Jaafar [1], Hendrik Wollmerstaedt [3], Parvaneh Rahimi [1], Helko Borsdorf [2], Stefan Zimmermann [4] and Yvonne Joseph [1]

1 Institute of Electronic and Sensor Materials, Faculty of Materials Science and Materials Technology, TU Bergakademie Freiberg, 09599 Freiberg, Germany; Alaa.Jaafar@doktorand.tu-freiberg.de (A.J.), Parvaneh.Rahimi@esm.tu-freiberg.de (P.R.); Yvonne.Joseph@esm.tu-freiberg.de (Y.J.)

2 UFZ-Helmholtz Centre for Environmental Research, Department Monitoring and Exploration Technologies, 04318 Leipzig, Germany; helko.borsdorf@ufz.de

3 Institute of Energy Process Engineering and Chemical Engineering, Chair of Reaction Engineering, Faculty of Mechanical, Process and Energy Engineering, TU Bergakademie Freiberg, 09599 Freiberg, Germany; Hendrik.Wollmerstaedt@iec.tu-freiberg.de

4 Institute of Electrical Engineering and Measurement Technology, Department of Sensors and Measurement Technology, Leibniz University Hannover, 30167 Hannover, Germany; zimmermann@geml.uni-hannover.de

* Correspondence: Mashaalah.Zarejousheghani@esm.tu-freiberg.de; Tel.: +49-3731-39-3598

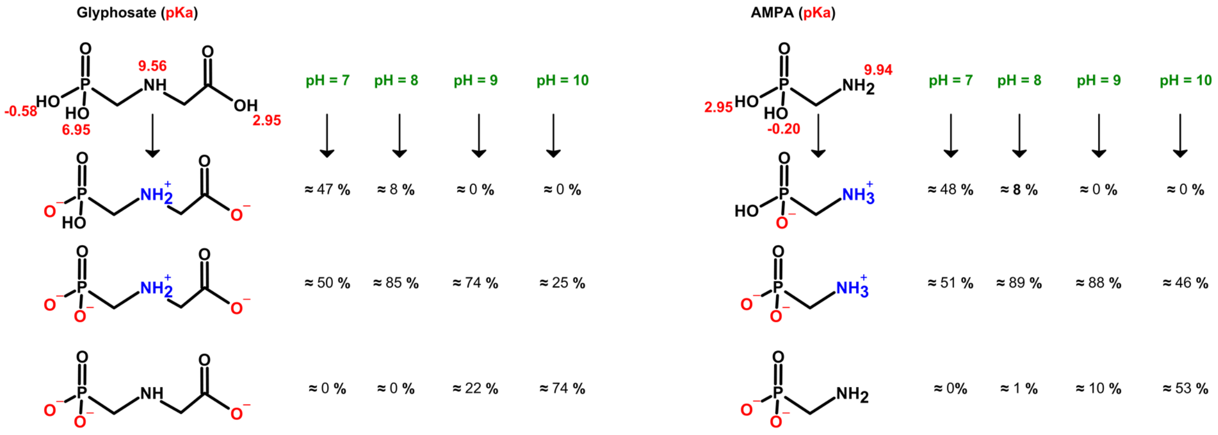

Figure S1. Different glyphosate and AMPA ions at different pHs (https://chemicalize.com)

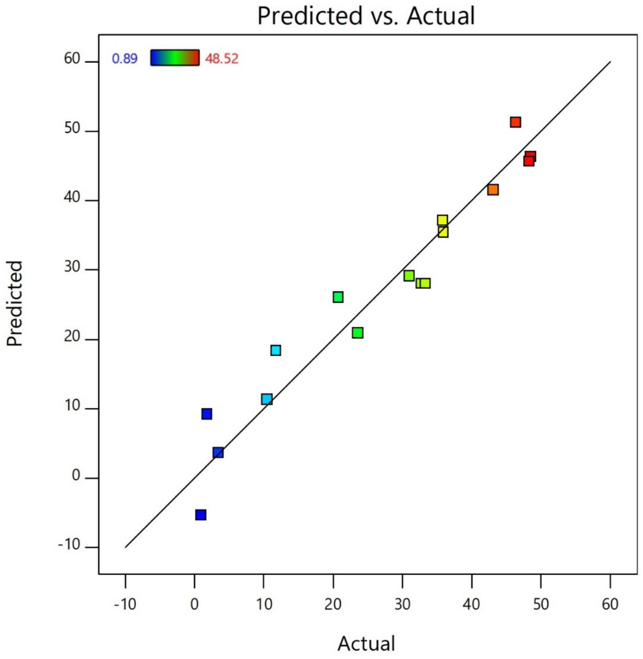

Figure S2. Actual vs Predicted plot for the developed model

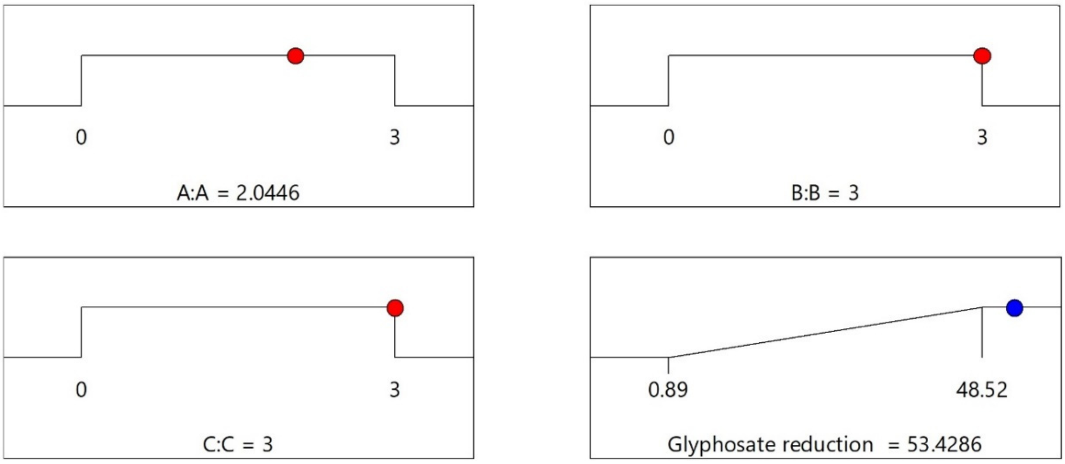

Figure S3. Desirability ramp of optimization.

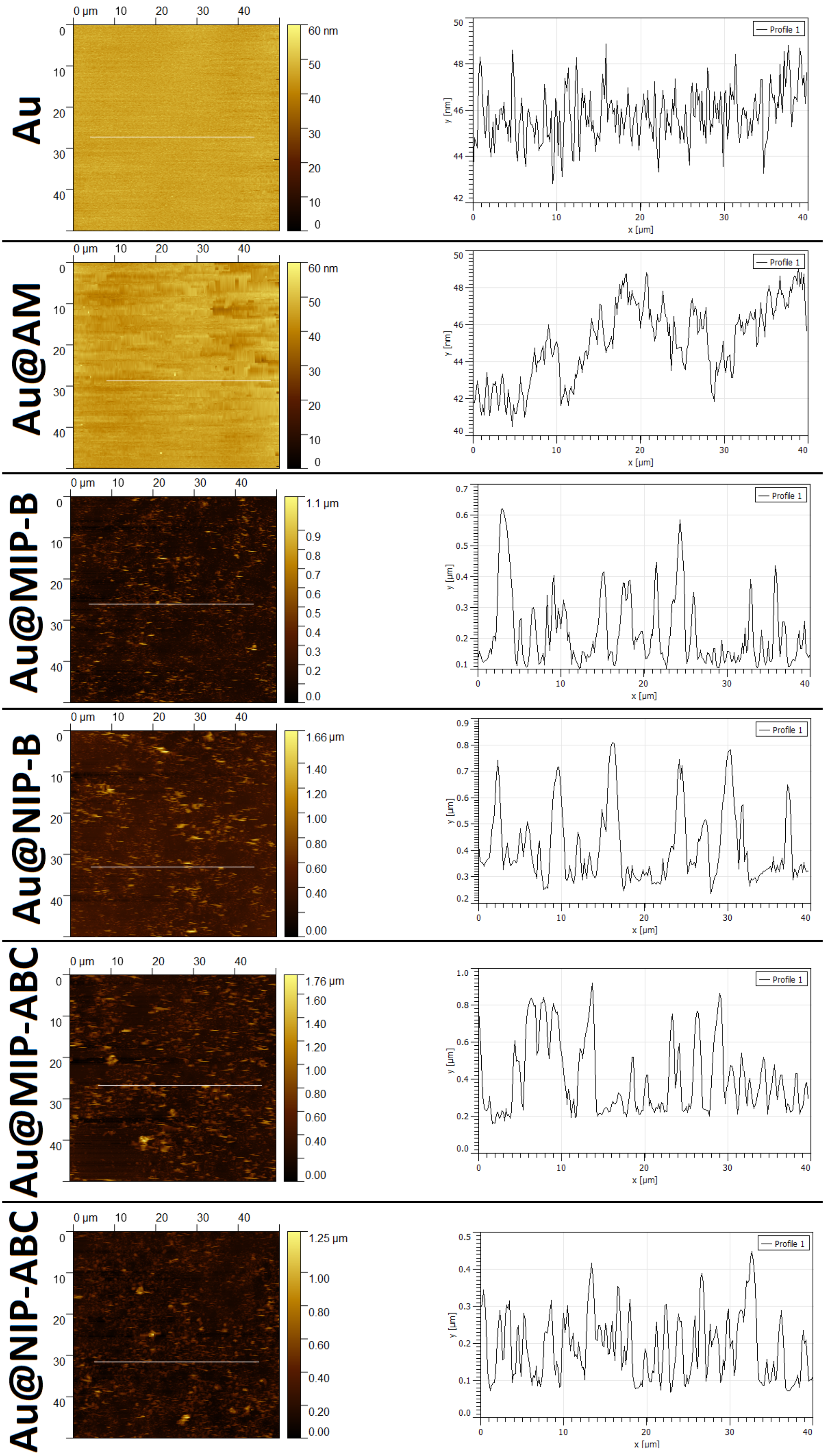

Figure S4. AFM 2D images and the height profiles for the corresponding lines drawn in AFM images

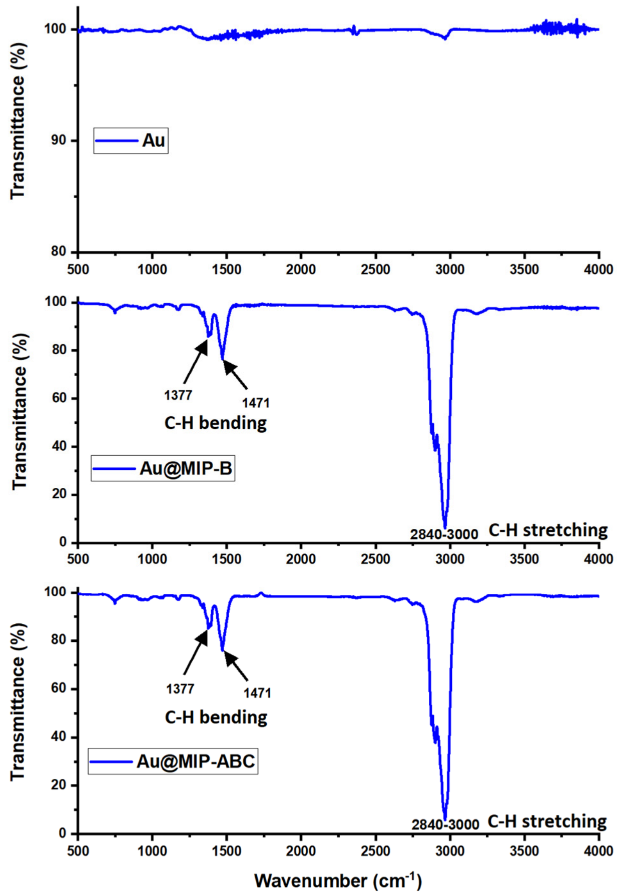

Figure S5. FTIR spectrums of the evaluated sensors

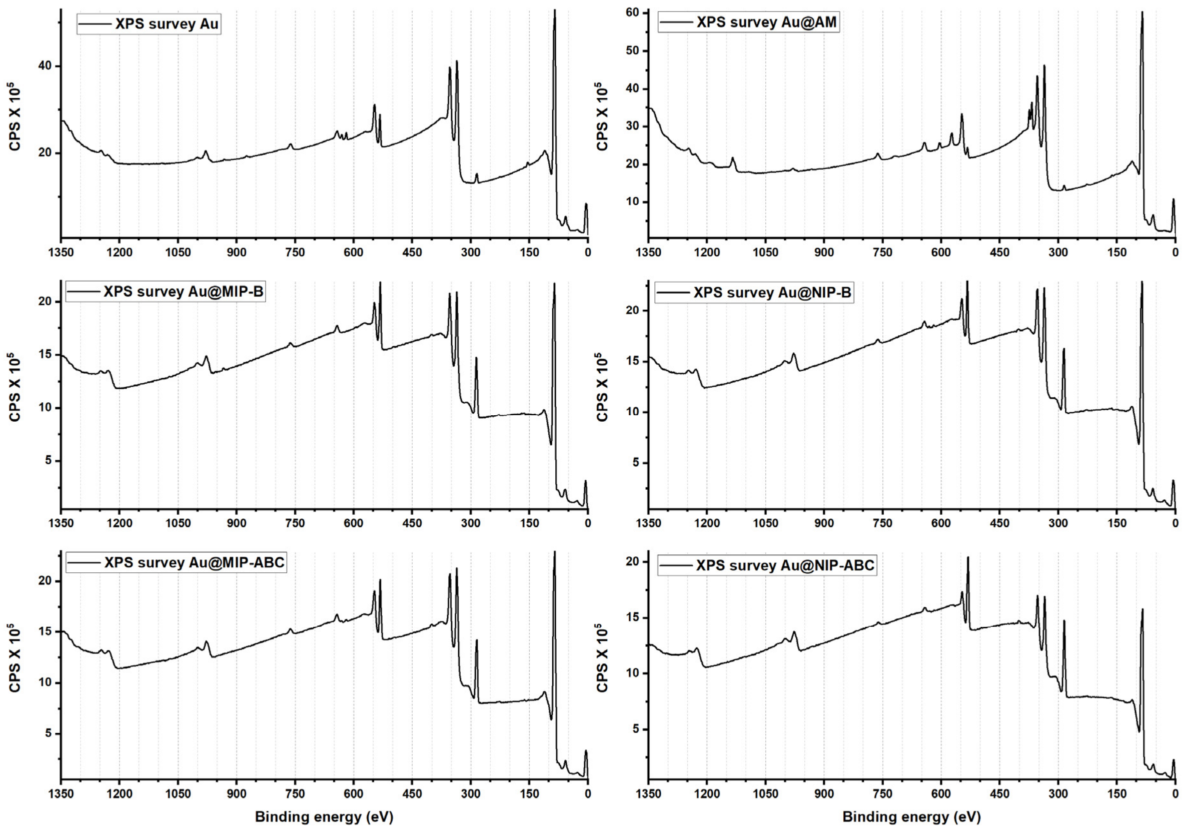

Figure S6. XPS survey spectra of all evaluated sensors (from 0 to 1350eV)

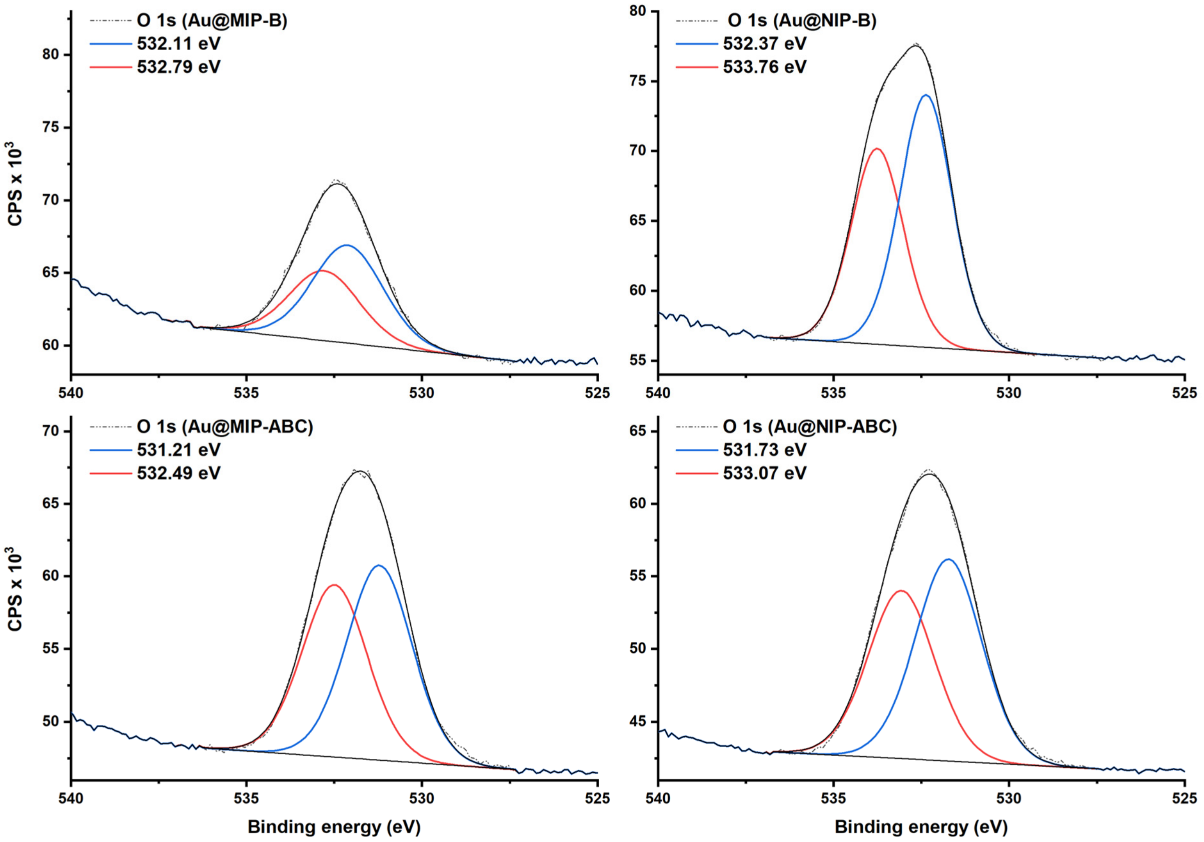

Figure S7. O 1s high-resolution XPS spectra of sensors modified with imprinted and non-imprinted polymers.
